# Crystal structure of 2-benzyl­amino-4-(4-meth­oxy­phen­yl)-6,7,8,9-tetra­hydro-5*H*-cyclo­hepta­[*b*]pyridine-3-carbo­nitrile

**DOI:** 10.1107/S1600536814024878

**Published:** 2014-11-19

**Authors:** R. A. Nagalakshmi, J. Suresh, S. Maharani, R. Ranjith Kumar, P. L. Nilantha Lakshman

**Affiliations:** a Department of Physics, The Madura College, Madurai 625 011, India; b Department of Organic Chemistry, School of Chemistry, Madurai Kamaraj University, Madurai 625 021, India; c Department of Food Science and Technology, University of Ruhuna, Mapalana, Kamburupitiya 81100, Sri Lanka

**Keywords:** crystal structure, cyclo­hepta­pyridine, carbo­nitrile, hydrogen bonding, C—H⋯π inter­actions, slipped parallel π–π inter­actions

## Abstract

The title compound, C_25_H_25_N_3_O, comprises a 2-amino­pyridine ring fused with a cyclo­heptane ring, which adopts a chair conformation. The central pyridine ring (r.m.s. deviation = 0.013 Å) carries three substituents, *viz.* a benzyl­amino group, a meth­oxy­phenyl ring and a carbo­nitrile group. The N atom of the carbo­nitrile group is significantly displaced [by 0.2247 (1) Å] from the plane of the pyridine ring, probably due to steric crowding involving the adjacent substituents. The phenyl and benzene rings are inclined to one another by 58.91 (7)° and to the pyridine ring by 76.68 (7) and 49.80 (6)°, respectively. In the crystal, inversion dimers linked by pairs of N—H⋯N_nitrile_ hydrogen bonds generate *R*_2_^2^(14) loops. The dimers are linked by C—H⋯π and slipped parallel π–π inter­actions [centroid–centroid distance = 3.6532 (3) Å] into a three-dimensional structure.

## Abstract 2

The title compound comprises a 2-amino­pyridine ring fused with a cyclo­heptane ring, which adopts a chair conformation. In the crystal, mol­ecules are linked *via* pairs of N—H⋯N_nitrile_ hydrogen bonds, forming inversion dimers which enclose 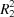
(14) ring motifs

## Chemical context   

The pyridine nucleus is prevalent in numerous natural products and extremely important in the chemistry of bio­log­ical systems (Bringmann *et al.*, 2004 ▶). 3-Cyano­pyridine or pyridine-3-carbo­nitrile derivatives attract particular attention for their wide-spectrum biological activity along with their importance and utility as inter­mediates in the preparation of a variety of heterocyclic compounds (Shishoo *et al.*, 1983 ▶; Doe *et al.*, 1990 ▶). 3-Cyano­pyridines with different alkyl and ar­yl/heteroaryl groups have been found to have a number of biological properties including anti­tubercular, anti­microbial, anti­cancer, A2A adenosine receptor antagonists (Mantri *et al.*, 2008 ▶), anti­hypertensive (Krauze *et al.*, 1985 ▶), anti­histaminic (Quintela *et al.*, 1997 ▶), anti-inflammatory, analgesic and anti­pyretic (Manna *et al.*, 1999 ▶) properties. Our inter­est in the preparation of pharmacologically active 3-cyano­pyridines led us to synthesise the title compound and the X-ray crystal structure determination was undertaken in order to establish its conformation.
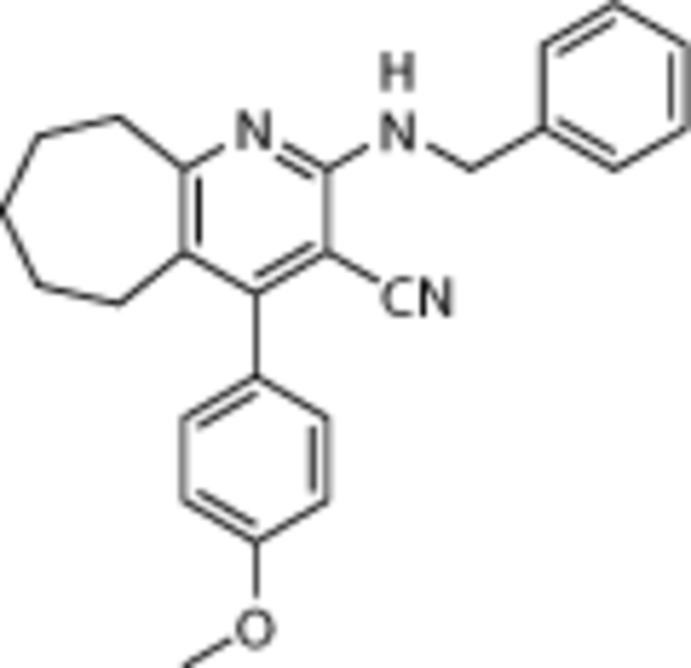


## Structural commentary   

**Figure 1:**
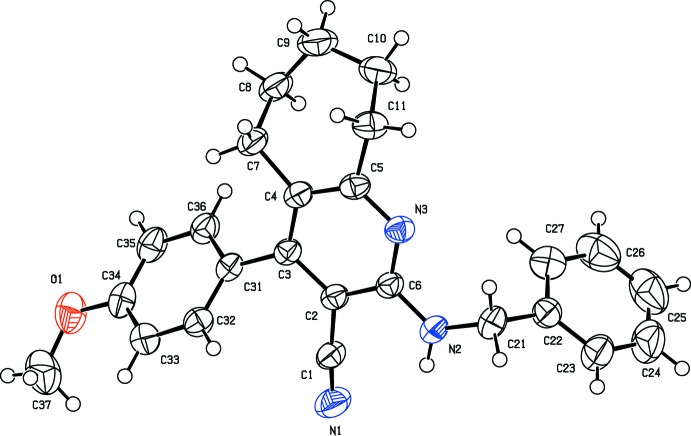
The mol­ecular structure of the title compound, showing the atom labelling. Displacement ellipsoids are drawn at the 50% probability level.

The mol­ecular structure of the title compound is shown in Fig. 1 ▶. The pyridine ring is connected to a benzene ring by a –CH_2_—NH_2_– chain. The cyclo­heptane ring adopts a chair conformation with puckering parameters *Q*2 = 0.4634 (15) Å, ϕ2 = 304.24 (18)° and *Q*3 = 0.6481 (16) Å and ϕ3 = 284.37 (12)°. The phenyl (C22–C27) and benzene (C31–C36) rings are inclined to one another by 58.91 (7)° and to the pyridine (N3/C2–C6) ring by 76.68 (7) and 49.80 (6)°, respectively. The N atom of the carbo­nitrile group, N1, is significantly displaced by 0.2247 (1) Å from the plane of the pyridine ring, perhaps due to steric crowding. The shortening of the C—N distance [C5—N3 = 1.3390 (14) Å] and the opening of the N3—C5—C4 angle to 124.47 (10)° may be attributed to the size of the substituent at C1, and correlates well with the values observed in a similar structure (Çelik *et al.*, 2013 ▶). There is conjugation between the donor (NH) and the acceptor (CN) groups *via* the C2—C6 bond. Thus the C6—N2 distance of 1.3502 (14) Å is shorter than the average conjugated C—N single bond, 1.370 (1) Å, found in the Cambridge Structural Database (Version 5.35; Groom & Allen, 2014 ▶). Steric hindrances rotate the benzene ring out of the plane of the central pyridine ring by 49.80 (6)°. This twist may be due to the non-bonded inter­actions between one of the *ortho* H atoms of the benzene ring and atom H7*B* of the cyclo­heptane ring. As a result of the π–π conjugation of atom O1, the O1—C34 bond length of 1.3618 (15) Å is significantly shorter than the O1—C37 distance of 1.410 (2) Å. An enlarge­ment of bond angle [C33—C34—O1 = 124.34 (13)°] on one side and a narrowing of bond angle [C35—C34—O1 = 116.29 (12)°] on the other side of the benzene ring may be due to the steric repulsion between the aromatic rings and the methyl group, as found in a similar structure (Tokuno *et al.*, 1986 ▶).

## Supra­molecular features   

**Figure 2:**
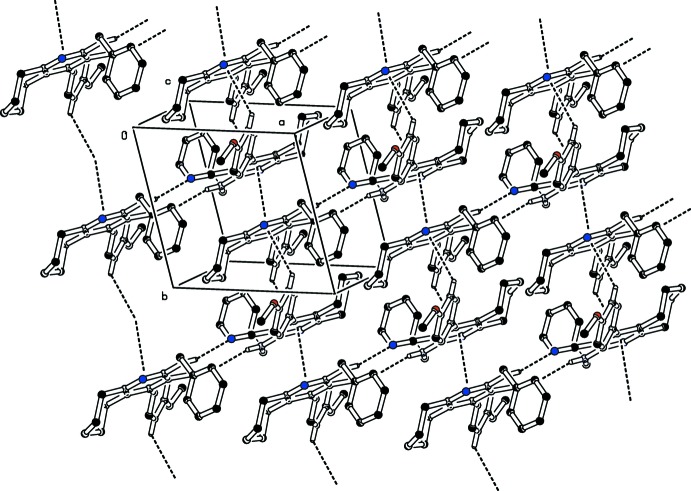
Partial packing diagram for the title compound, viewed along the *c* axis. Dashed lines represent inter­molecular hydrogen bonds and C—H⋯π contacts (see Table 1 ▶ for details; H atoms not involved in hydrogen bonding have been omitted for clarity).

In the crystal, mol­ecules are linked *via* pairs of N—H⋯N_nitrile_ inter­actions, forming inversion dimers which enclose 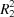
(14) ring motifs. The dimers are connected through weak C—H⋯π inter­actions involving the CN group as acceptor (Table 1 ▶). They are further connected by slipped parallel π–π stacking inter­actions involving the pyridine rings of inversion-related mol­ecules [*Cg*1⋯*Cg*1^i^ = 3.6532 (7), normal distance = 3.5920 (5), slippage = 0.667 Å; *Cg*1 is the centroid of the N3/C2–C6 ring; symmetry code: (i) −*x* + 1, −*y* + 1, −*z* + 1], as shown in Fig. 2 ▶.

## Database survey   

In the title compound, the chair conformation of the cyclo­octane ring and the planar conformation of the pyridine are similar to those found in the related structure 2-(4-bromo­phen­yl)-4-(4-meth­oxy­phen­yl)-6,7,8,9-tetra­hydro-5*H*-cyclohepta­[*b*]pyridine (Çelik *et al.*, 2013 ▶). However, the C6—N2H and C1 N1 groups whose presence in the title compound allows the formation of N—H⋯N hydrogen bonds, are not present in the above-cited compound. In the title compound, C—C bonds involving atom C2, which is substituted by the C1 N1 group [C2—C3 = 1.4024 (15) and C2—C6 = 1.4076 (16) Å] are systematically longer than those found in the related structure [1.392 (4) and 1.378 (4) Å, respectively]. In the title compound, steric hindrance rotates the 4-meth­oxy­phenyl ring (C31–C36) and the phenyl ring (C22–C27), which are inclined to the plane of the central pyridine ring by 49.80 (6) and 76.68 (7)°, respectively. In the related structure (Çelik *et al.*, 2013 ▶), the 4-bromo­phenyl ring is almost coplanar with the pyridine ring, making a dihedral angle of 8.27 (16)° while the 4-meth­oxy­phenyl ring is inclined to the pyridine ring by 58.63 (15)°, compared with 49.80 (6)° in the title compound.

## Synthesis and crystallization   

A mixture of cyclo­hepta­none (1 mmol), 4-meth­oxy aldehyde (1 mmol) and malono­nitrile (1 mmol) and benzyl­amine (1mmol) was taken in ethanol (10 ml) to which *p*-TSA (1.0 mmol) was added. The reaction mixture was heated under reflux for 2–3 h. On completion of the reaction, checked by thin-layer chromatography (TLC), the mixture was poured into crushed ice and extracted with ethyl acetate. The excess solvent was removed under vacuum and the residue was subjected to column chromatography using petroleum ether/ethyl acetate mixture (97:3 *v*/*v*) as eluent to afford pure product. The product was recrystallized from ethyl acetate, affording colourless crystals of the title compound. (m.p. 415 K; yield 75%).

## Refinement   

Crystal data, data collection and structure refinement details are summarized in Table 2 ▶. The NH and C-bound H atoms were placed in calculated positions and allowed to ride on their carrier atoms: N—H = 0.86 and C—H = 0.93–0.97 Å, with *U*_iso_(H) = 1.5*U*_eq_(C) for methyl H atoms and 1.2*U*_eq_(N,C) for other H atoms. The DELU restraint was applied.

## Supplementary Material

Crystal structure: contains datablock(s) global, I. DOI: 10.1107/S1600536814024878/su5014sup1.cif

Structure factors: contains datablock(s) I. DOI: 10.1107/S1600536814024878/su5014Isup2.hkl

Supporting information file. DOI: 10.1107/S1600536814024878/su5014Isup3.cml

CCDC reference: 1033842

Additional supporting information:  crystallographic information; 3D view; checkCIF report

## Figures and Tables

**Table 1:** Hydrogen-bond geometry (, ) *Cg*1 is the centroid of pyridine ring N3/C2C6.

*D*H*A*	*D*H	H*A*	*D* *A*	*D*H*A*
N2H2N1^i^	0.86	2.28	3.0168(15)	145
C35H35*Cg*1^ii^	0.93	2.99	3.5559(14)	121

Symmetry codes: (i) 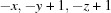; (ii) 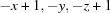.

**Table 2:** Experimental details

Crystal data	
Chemical formula	C_25_H_25_N_3_O
*M*_r_	383.48
Crystal system, space group	Triclinic, *P* 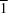
Temperature (K)	293
*a*, *b*, *c* ()	8.8509(2), 9.6364(3), 12.9090(4)
, , ()	72.779(2), 81.033(1), 76.457(1)
*V* (^3^)	1017.97(5)
*Z*	2
Radiation type	Mo *K*
(mm^1^)	0.08
Crystal size (mm)	0.21 0.19 0.18

Data collection	
Diffractometer	Bruker Kappa APEXII
Absorption correction	Multi-scan (*SADABS*; Bruker, 2004 ▶)
*T*_min_, *T*_max_	0.967, 0.974
No. of measured, independent and observed [*I* > 2(*I*)] reflections	22986, 3798, 3177
*R*_int_	0.023
(sin /)_max_ (^1^)	0.606

Refinement	
*R*[*F* ^2^ > 2(*F* ^2^)], *wR*(*F* ^2^), *S*	0.035, 0.096, 1.05
No. of reflections	3798
No. of parameters	263
No. of restraints	1
H-atom treatment	H-atom parameters constrained
_max_, _min_ (e ^3^)	0.14, 0.13

Computer programs: *APEX2* and *SAINT* (Bruker, 2004 ▶), *SHELXS97* and *SHELXL2014* (Sheldrick, 2008 ▶) and *PLATON* (Spek, 2009 ▶).

## supplementary crystallographic information

### Crystal data

**Table:**

C_25_H_25_N_3_O	*Z* = 2
*M**_r_* = 383.48	*F*(000) = 408
Triclinic, *P*1	*D*_x_ = 1.251 Mg m^−^^3^
*a* = 8.8509 (2) Å	Mo *K*α radiation, λ = 0.71073 Å
*b* = 9.6364 (3) Å	Cell parameters from 2000 reflections
*c* = 12.9090 (4) Å	θ = 2–31°
α = 72.779 (2)°	µ = 0.08 mm^−^^1^
β = 81.033 (1)°	*T* = 293 K
γ = 76.457 (1)°	Block, colourless
*V* = 1017.97 (5) Å^3^	0.21 × 0.19 × 0.18 mm

### Data collection

**Table:**

Bruker Kappa APEXII diffractometer	3177 reflections with *I* > 2σ(*I*)
Radiation source: fine-focus sealed tube	*R*_int_ = 0.023
φ & ω scans	θ_max_ = 25.5°, θ_min_ = 2.3°
Absorption correction: multi-scan (*SADABS*; Bruker, 2004)	*h* = −10→10
*T*_min_ = 0.967, *T*_max_ = 0.974	*k* = −11→11
22986 measured reflections	*l* = −15→15
3798 independent reflections	

### Refinement

**Table:**

Refinement on *F*^2^	Hydrogen site location: inferred from neighbouring sites
Least-squares matrix: full	H-atom parameters constrained
*R*[*F*^2^ > 2σ(*F*^2^)] = 0.035	*w* = 1/[σ^2^(*F*_o_^2^) + (0.0427*P*)^2^ + 0.190*P*] where *P* = (*F*_o_^2^ + 2*F*_c_^2^)/3
*wR*(*F*^2^) = 0.096	(Δ/σ)_max_ < 0.001
*S* = 1.05	Δρ_max_ = 0.14 e Å^−^^3^
3798 reflections	Δρ_min_ = −0.13 e Å^−^^3^
263 parameters	Extinction correction: *SHELXL2014* (Sheldrick, 2008), Fc^*^=kFc[1+0.001xFc^2^λ^3^/sin(2θ)]^-1/4^
1 restraint	Extinction coefficient: 0.028 (3)

### Special details

**Table:**

Geometry. All e.s.d.'s (except the e.s.d. in the dihedral angle between two l.s. planes) are estimated using the full covariance matrix. The cell e.s.d.'s are taken into account individually in the estimation of e.s.d.'s in distances, angles and torsion angles; correlations between e.s.d.'s in cell parameters are only used when they are defined by crystal symmetry. An approximate (isotropic) treatment of cell e.s.d.'s is used for estimating e.s.d.'s involving l.s. planes.

### Fractional atomic coordinates and isotropic or equivalent isotropic displacement parameters (Å^2^)

**Table:**

	*x*	*y*	*z*	*U*_iso_*/*U*_eq_	
C1	0.22942 (13)	0.36297 (13)	0.46578 (10)	0.0409 (3)	
C2	0.36435 (12)	0.33965 (12)	0.52093 (9)	0.0347 (3)	
C3	0.51117 (12)	0.26601 (11)	0.48631 (9)	0.0339 (3)	
C4	0.63263 (12)	0.23924 (12)	0.55100 (9)	0.0365 (3)	
C5	0.60042 (13)	0.28670 (12)	0.64557 (10)	0.0375 (3)	
C6	0.34347 (13)	0.38756 (12)	0.61609 (9)	0.0356 (3)	
C7	0.79452 (13)	0.15737 (14)	0.52501 (11)	0.0445 (3)	
H7A	0.8695	0.2157	0.5264	0.053*	
H7B	0.8032	0.1463	0.4519	0.053*	
C8	0.83483 (15)	0.00402 (15)	0.60507 (12)	0.0528 (3)	
H8A	0.7422	−0.0390	0.6253	0.063*	
H8B	0.9128	−0.0595	0.5689	0.063*	
C9	0.89589 (17)	0.00700 (17)	0.70713 (13)	0.0653 (4)	
H9A	0.9892	0.0490	0.6864	0.078*	
H9B	0.9259	−0.0942	0.7509	0.078*	
C10	0.78190 (17)	0.09421 (16)	0.77684 (12)	0.0589 (4)	
H10A	0.8313	0.0874	0.8406	0.071*	
H10B	0.6919	0.0477	0.8018	0.071*	
C11	0.72484 (15)	0.25800 (14)	0.72061 (11)	0.0495 (3)	
H11A	0.6845	0.3103	0.7759	0.059*	
H11B	0.8132	0.2987	0.6791	0.059*	
C21	0.16754 (15)	0.51651 (14)	0.74336 (11)	0.0471 (3)	
H21A	0.1077	0.6172	0.7243	0.057*	
H21B	0.2642	0.5190	0.7687	0.057*	
C22	0.07695 (14)	0.42293 (14)	0.83428 (10)	0.0438 (3)	
C23	−0.06616 (17)	0.48310 (19)	0.87858 (13)	0.0618 (4)	
H23	−0.1098	0.5826	0.8503	0.074*	
C24	−0.1451 (2)	0.3967 (3)	0.96458 (15)	0.0812 (5)	
H24	−0.2414	0.4385	0.9943	0.097*	
C25	−0.0834 (2)	0.2504 (3)	1.00645 (14)	0.0873 (6)	
H25	−0.1366	0.1929	1.0650	0.105*	
C26	0.0576 (2)	0.1889 (2)	0.96175 (15)	0.0814 (5)	
H26	0.0996	0.0888	0.9893	0.098*	
C27	0.13711 (18)	0.27461 (17)	0.87634 (12)	0.0608 (4)	
H27	0.2329	0.2319	0.8465	0.073*	
C31	0.52942 (12)	0.22205 (12)	0.38341 (9)	0.0358 (3)	
C32	0.47550 (14)	0.32373 (13)	0.29000 (10)	0.0411 (3)	
H32	0.4308	0.4205	0.2920	0.049*	
C33	0.48591 (15)	0.28606 (14)	0.19354 (10)	0.0464 (3)	
H33	0.4488	0.3569	0.1317	0.056*	
C34	0.55151 (14)	0.14306 (15)	0.18936 (11)	0.0457 (3)	
C35	0.60779 (15)	0.03992 (14)	0.28153 (11)	0.0494 (3)	
H35	0.6533	−0.0565	0.2790	0.059*	
C36	0.59703 (14)	0.07863 (13)	0.37677 (10)	0.0432 (3)	
H36	0.6356	0.0078	0.4382	0.052*	
C37	0.4929 (2)	0.1922 (2)	0.00758 (13)	0.0789 (5)	
H37A	0.5107	0.1452	−0.0505	0.118*	
H37B	0.3827	0.2179	0.0267	0.118*	
H37C	0.5360	0.2803	−0.0158	0.118*	
N1	0.11429 (13)	0.38506 (15)	0.42880 (11)	0.0609 (3)	
N2	0.20363 (12)	0.46344 (12)	0.64721 (8)	0.0466 (3)	
H2	0.1297	0.4817	0.6061	0.056*	
N3	0.46071 (11)	0.35839 (10)	0.67815 (8)	0.0389 (2)	
O1	0.56525 (13)	0.09400 (12)	0.09900 (8)	0.0639 (3)	

### Atomic displacement parameters (Å^2^)

**Table:**

	*U*^11^	*U*^22^	*U*^33^	*U*^12^	*U*^13^	*U*^23^
C1	0.0330 (6)	0.0428 (7)	0.0465 (7)	−0.0009 (5)	−0.0026 (5)	−0.0170 (5)
C2	0.0319 (5)	0.0311 (5)	0.0385 (6)	−0.0031 (4)	−0.0034 (4)	−0.0082 (5)
C3	0.0323 (6)	0.0271 (5)	0.0391 (6)	−0.0052 (4)	−0.0001 (5)	−0.0062 (4)
C4	0.0305 (6)	0.0319 (6)	0.0435 (7)	−0.0044 (4)	−0.0015 (5)	−0.0070 (5)
C5	0.0348 (6)	0.0307 (6)	0.0449 (7)	−0.0055 (5)	−0.0063 (5)	−0.0068 (5)
C6	0.0329 (6)	0.0307 (6)	0.0392 (6)	−0.0025 (4)	−0.0018 (5)	−0.0072 (5)
C7	0.0293 (6)	0.0477 (7)	0.0522 (7)	−0.0033 (5)	−0.0003 (5)	−0.0122 (6)
C8	0.0375 (7)	0.0459 (7)	0.0657 (9)	0.0053 (5)	−0.0003 (6)	−0.0139 (6)
C9	0.0504 (8)	0.0565 (9)	0.0750 (10)	0.0083 (7)	−0.0177 (7)	−0.0054 (8)
C10	0.0580 (8)	0.0564 (8)	0.0558 (9)	0.0014 (7)	−0.0214 (7)	−0.0070 (7)
C11	0.0437 (7)	0.0491 (7)	0.0576 (8)	−0.0049 (6)	−0.0165 (6)	−0.0147 (6)
C21	0.0447 (7)	0.0457 (7)	0.0514 (8)	−0.0041 (5)	0.0035 (6)	−0.0218 (6)
C22	0.0416 (6)	0.0521 (7)	0.0424 (7)	−0.0079 (5)	−0.0061 (5)	−0.0198 (6)
C23	0.0473 (8)	0.0730 (10)	0.0633 (9)	−0.0085 (7)	0.0044 (7)	−0.0234 (8)
C24	0.0555 (9)	0.1198 (17)	0.0688 (11)	−0.0296 (10)	0.0128 (8)	−0.0266 (11)
C25	0.0843 (13)	0.1211 (17)	0.0583 (10)	−0.0573 (13)	−0.0036 (9)	0.0001 (11)
C26	0.0897 (13)	0.0735 (11)	0.0731 (11)	−0.0277 (10)	−0.0196 (10)	0.0070 (9)
C27	0.0603 (9)	0.0583 (9)	0.0593 (9)	−0.0069 (7)	−0.0090 (7)	−0.0114 (7)
C31	0.0298 (5)	0.0352 (6)	0.0416 (6)	−0.0066 (4)	0.0021 (5)	−0.0121 (5)
C32	0.0416 (6)	0.0363 (6)	0.0458 (7)	−0.0043 (5)	−0.0034 (5)	−0.0146 (5)
C33	0.0480 (7)	0.0489 (7)	0.0423 (7)	−0.0089 (6)	−0.0038 (5)	−0.0133 (6)
C34	0.0421 (7)	0.0531 (7)	0.0489 (7)	−0.0151 (6)	0.0079 (5)	−0.0262 (6)
C35	0.0503 (7)	0.0394 (7)	0.0584 (8)	−0.0057 (5)	0.0074 (6)	−0.0219 (6)
C36	0.0415 (6)	0.0364 (6)	0.0475 (7)	−0.0043 (5)	0.0033 (5)	−0.0117 (5)
C37	0.0974 (13)	0.0970 (13)	0.0560 (10)	−0.0224 (11)	−0.0081 (9)	−0.0384 (10)
N1	0.0385 (6)	0.0780 (9)	0.0695 (8)	0.0018 (6)	−0.0126 (6)	−0.0316 (7)
N2	0.0364 (5)	0.0562 (6)	0.0435 (6)	0.0055 (5)	−0.0036 (4)	−0.0196 (5)
N3	0.0380 (5)	0.0348 (5)	0.0433 (6)	−0.0025 (4)	−0.0067 (4)	−0.0119 (4)
O1	0.0726 (7)	0.0731 (7)	0.0567 (6)	−0.0153 (5)	0.0037 (5)	−0.0381 (5)

### Geometric parameters (Å, º)

**Table:**

C1—N1	1.1407 (16)	C21—H21B	0.9700
C1—C2	1.4242 (16)	C22—C23	1.3763 (19)
C2—C3	1.4024 (15)	C22—C27	1.377 (2)
C2—C6	1.4076 (16)	C23—C24	1.377 (2)
C3—C4	1.3935 (16)	C23—H23	0.9300
C3—C31	1.4848 (16)	C24—C25	1.363 (3)
C4—C5	1.3935 (17)	C24—H24	0.9300
C4—C7	1.5052 (15)	C25—C26	1.368 (3)
C5—N3	1.3390 (14)	C25—H25	0.9300
C5—C11	1.5029 (16)	C26—C27	1.373 (2)
C6—N3	1.3367 (15)	C26—H26	0.9300
C6—N2	1.3502 (14)	C27—H27	0.9300
C7—C8	1.5307 (17)	C31—C32	1.3794 (17)
C7—H7A	0.9700	C31—C36	1.3936 (16)
C7—H7B	0.9700	C32—C33	1.3809 (17)
C8—C9	1.510 (2)	C32—H32	0.9300
C8—H8A	0.9700	C33—C34	1.3778 (18)
C8—H8B	0.9700	C33—H33	0.9300
C9—C10	1.517 (2)	C34—O1	1.3618 (15)
C9—H9A	0.9700	C34—C35	1.3800 (19)
C9—H9B	0.9700	C35—C36	1.3704 (18)
C10—C11	1.5283 (18)	C35—H35	0.9300
C10—H10A	0.9700	C36—H36	0.9300
C10—H10B	0.9700	C37—O1	1.410 (2)
C11—H11A	0.9700	C37—H37A	0.9600
C11—H11B	0.9700	C37—H37B	0.9600
C21—N2	1.4422 (16)	C37—H37C	0.9600
C21—C22	1.5007 (18)	N2—H2	0.8600
C21—H21A	0.9700		
			
N1—C1—C2	174.35 (13)	C22—C21—H21B	108.9
C3—C2—C6	120.54 (10)	H21A—C21—H21B	107.7
C3—C2—C1	122.31 (10)	C23—C22—C27	118.53 (13)
C6—C2—C1	117.08 (10)	C23—C22—C21	121.14 (12)
C4—C3—C2	117.51 (10)	C27—C22—C21	120.32 (12)
C4—C3—C31	123.69 (10)	C22—C23—C24	120.32 (16)
C2—C3—C31	118.80 (10)	C22—C23—H23	119.8
C3—C4—C5	118.10 (10)	C24—C23—H23	119.8
C3—C4—C7	122.90 (11)	C25—C24—C23	120.59 (17)
C5—C4—C7	118.97 (10)	C25—C24—H24	119.7
N3—C5—C4	124.47 (10)	C23—C24—H24	119.7
N3—C5—C11	114.61 (11)	C24—C25—C26	119.55 (16)
C4—C5—C11	120.92 (10)	C24—C25—H25	120.2
N3—C6—N2	118.40 (10)	C26—C25—H25	120.2
N3—C6—C2	121.02 (10)	C25—C26—C27	120.14 (17)
N2—C6—C2	120.58 (10)	C25—C26—H26	119.9
C4—C7—C8	112.62 (10)	C27—C26—H26	119.9
C4—C7—H7A	109.1	C26—C27—C22	120.86 (15)
C8—C7—H7A	109.1	C26—C27—H27	119.6
C4—C7—H7B	109.1	C22—C27—H27	119.6
C8—C7—H7B	109.1	C32—C31—C36	117.32 (11)
H7A—C7—H7B	107.8	C32—C31—C3	120.14 (10)
C9—C8—C7	113.46 (12)	C36—C31—C3	122.52 (11)
C9—C8—H8A	108.9	C31—C32—C33	121.92 (11)
C7—C8—H8A	108.9	C31—C32—H32	119.0
C9—C8—H8B	108.9	C33—C32—H32	119.0
C7—C8—H8B	108.9	C34—C33—C32	119.68 (12)
H8A—C8—H8B	107.7	C34—C33—H33	120.2
C8—C9—C10	115.00 (11)	C32—C33—H33	120.2
C8—C9—H9A	108.5	O1—C34—C33	124.34 (13)
C10—C9—H9A	108.5	O1—C34—C35	116.29 (12)
C8—C9—H9B	108.5	C33—C34—C35	119.38 (12)
C10—C9—H9B	108.5	C36—C35—C34	120.43 (11)
H9A—C9—H9B	107.5	C36—C35—H35	119.8
C9—C10—C11	115.37 (13)	C34—C35—H35	119.8
C9—C10—H10A	108.4	C35—C36—C31	121.27 (12)
C11—C10—H10A	108.4	C35—C36—H36	119.4
C9—C10—H10B	108.4	C31—C36—H36	119.4
C11—C10—H10B	108.4	O1—C37—H37A	109.5
H10A—C10—H10B	107.5	O1—C37—H37B	109.5
C5—C11—C10	114.29 (11)	H37A—C37—H37B	109.5
C5—C11—H11A	108.7	O1—C37—H37C	109.5
C10—C11—H11A	108.7	H37A—C37—H37C	109.5
C5—C11—H11B	108.7	H37B—C37—H37C	109.5
C10—C11—H11B	108.7	C6—N2—C21	125.66 (11)
H11A—C11—H11B	107.6	C6—N2—H2	117.2
N2—C21—C22	113.29 (10)	C21—N2—H2	117.2
N2—C21—H21A	108.9	C6—N3—C5	118.31 (10)
C22—C21—H21A	108.9	C34—O1—C37	117.32 (12)
N2—C21—H21B	108.9		
			
C6—C2—C3—C4	1.68 (15)	C23—C24—C25—C26	−0.7 (3)
C1—C2—C3—C4	−175.20 (10)	C24—C25—C26—C27	0.9 (3)
C6—C2—C3—C31	−177.87 (10)	C25—C26—C27—C22	−0.1 (3)
C1—C2—C3—C31	5.26 (16)	C23—C22—C27—C26	−1.1 (2)
C2—C3—C4—C5	0.20 (15)	C21—C22—C27—C26	177.95 (14)
C31—C3—C4—C5	179.73 (10)	C4—C3—C31—C32	−130.14 (12)
C2—C3—C4—C7	178.13 (10)	C2—C3—C31—C32	49.38 (14)
C31—C3—C4—C7	−2.35 (17)	C4—C3—C31—C36	51.31 (16)
C3—C4—C5—N3	−0.95 (17)	C2—C3—C31—C36	−129.17 (12)
C7—C4—C5—N3	−178.96 (10)	C36—C31—C32—C33	0.64 (17)
C3—C4—C5—C11	178.41 (10)	C3—C31—C32—C33	−177.98 (10)
C7—C4—C5—C11	0.40 (16)	C31—C32—C33—C34	0.14 (19)
C3—C2—C6—N3	−3.01 (16)	C32—C33—C34—O1	178.99 (11)
C1—C2—C6—N3	174.02 (10)	C32—C33—C34—C35	−0.84 (18)
C3—C2—C6—N2	177.46 (10)	O1—C34—C35—C36	−179.08 (11)
C1—C2—C6—N2	−5.51 (16)	C33—C34—C35—C36	0.76 (19)
C3—C4—C7—C8	−109.94 (13)	C34—C35—C36—C31	0.04 (19)
C5—C4—C7—C8	67.96 (14)	C32—C31—C36—C35	−0.73 (17)
C4—C7—C8—C9	−84.84 (14)	C3—C31—C36—C35	177.86 (11)
C7—C8—C9—C10	62.48 (17)	N3—C6—N2—C21	−0.36 (18)
C8—C9—C10—C11	−59.44 (18)	C2—C6—N2—C21	179.18 (11)
N3—C5—C11—C10	113.46 (13)	C22—C21—N2—C6	−102.67 (14)
C4—C5—C11—C10	−65.96 (16)	N2—C6—N3—C5	−178.20 (10)
C9—C10—C11—C5	78.27 (16)	C2—C6—N3—C5	2.26 (16)
N2—C21—C22—C23	−122.40 (13)	C4—C5—N3—C6	−0.30 (16)
N2—C21—C22—C27	58.62 (16)	C11—C5—N3—C6	−179.69 (10)
C27—C22—C23—C24	1.3 (2)	C33—C34—O1—C37	−6.89 (19)
C21—C22—C23—C24	−177.67 (14)	C35—C34—O1—C37	172.95 (13)
C22—C23—C24—C25	−0.5 (3)		

### Hydrogen-bond geometry (Å, º)

Cg1 is the centroid of pyridine ring N3/C2–C6.

**Table:**

*D*—H···*A*	*D*—H	H···*A*	*D*···*A*	*D*—H···*A*
N2—H2···N1^i^	0.86	2.28	3.0168 (15)	145
C35—H35···*Cg*1^ii^	0.93	2.99	3.5559 (14)	121

Symmetry codes: (i) −*x*, −*y*+1, −*z*+1; (ii) −*x*+1, −*y*, −*z*+1.
